# Address on Metamorphosis and Metempsychosis

**Published:** 1854-05

**Authors:** 


ART. V.—*Address introductory to the Course of Lectures in the Chemical Department of the Vermont Medical College, delivered before the Class of Session* 1854. By. THOMAS ANTISELL, M. D., Professor of Chemistry to the College, and to the Berkshire Medical Institution. Published by the Class of the College. Woodstock; Press of the Vermont Temperance Standard; 1854.

---

The subject of this lecture is "*Metamorphosis and Metempsychosis*," and the author presents in a striking and beautiful light some of the curious and wonderful phenomena of chemical action. It is an address of rare excellence, abounding in facts, and impressed by the true spirit of philosophy and poetry. We cite a few passages in proof. The author takes, as an example of chemical action, or metamorphosis, a mineral spring, issuing from the crevices of the inner and heated part of the earth, and pursues the mutations of one of its constituents.

"These bubbles of fixed air, or carbonic acid, are made up of charcoal and the vivifying part of our atmosphere, oxygen. This gas, coy, aerial in its form, yet has earthly longings, and loves to creep close to the bosom of the earth, and twines within the deepest folds and wrinkles which Time has made upon her surface; bathes itself in the placid stream, and with it floats along, until it is carried to the sea, where it descends from its exalted position, and seats itself upon the billows of heaving ocean, and mingling in the foam and the waves, it pierces several fathoms deep, to the ocean bottom. There, on the dark slopes of a subaqueous hill, lie in wait myriads of beings, little sacs of membrane, invisible to the unaided eye; they twist their tiny arms about, and catch a bubble, with its coating of water, and greedily devour it; the water they return, the bubble is retained; presently a little limestone coat or shell is formed by the animal, and half the amount in weight is caused by this imprisonment and solidification of the gas. These little animalcules work and build together, and by numbers illimitable and succession continuous, they raise their limestone to the water level,—and behold, the coral reef. Trace it emerge steadily above the water, and the yam and the bread-fruit, the cocoa-nut and the screw-pine clothe its flat and whitened beaches with food and shelter for the wayfaring navigator or current-drifted Polynesian. Here is the restless activity of the atom. Vomited forth from the earth, it emerges into air—sinks down into the water—passes into the body of an animal, and becomes an integrant part thereof; and when the latter dies, it remains behind to form the basis of a durable rock. It is the real proteus; with its change of place it has changed its form;—in the air a gas, in the water a liquid, in the animal and the reef it is imprisoned as a solid. Nor is this a new office to it. Ever since that period in our planet's history, when the waters were gathered together into one place, has this carbonic acid fulfilled the same office, and built up, in the slimy depths of the ocean, these numerous mountain masses of limestone, which, by the movements of time and elevation, become the dry rock of the valleys around us. The labors of man fall into insignificance when compared with that of a minute animalcule, and less enduring monuments of his existence remain, than that of one of the smallest of God's creation.

"But some one will now say, your atom is now at rest. It is a solid rock, and has been so for ages. But are rocks eternal? Why does Nature's bosom throb and heave within continually, and seem almost ready to burst, until, as it were, no longer able to confine herself within the tight girdle that encircles her, she bursts the zone and raises up the land around us. What for? Because the limestone will not stay among us. It, too, has its office;—it yields itself to the rains of heaven, to the rivers of earth, and to the demands of growing vegetables, and the plant and tree draw it in for nutriment, and destroy its composition. The river yields it to the animals in drink, and the remainder is carried down and deposited as a fine chalk mud at the embouchure of the stream,—again, in the cycle of change, to be raised to dry land, and again to be removed. Where, then, is the period of rest? Shall we call the quiescence of even a series of ages in the life of an indestructible and an immortal, a permanent condition? It is rather delay than rest. We must not measure the periods of creation upon the Procrustes bed of our short lives; nor dare to imagine the duration of even the life of our species as more than a dot of sand in the hour-glass of eternity."

Again:—

"While nature thus performs her round of change in the great deeps, let me direct your attention to what is going on in the air. See yonder forest trees, with leaves glittering in the sunbeams, like the well polished armor of the serried ranks of infantry, busy drinking in illimitable volumes of this same fixed air, carrying it into its branches and meeting with the water sucked up by the roots, forming, by the union of this gas and water, the woody tissue, starch, and sugar. To accomplish it, the gas is decomposed, broken up into charcoal and oxygen; the oxygen is given off, the charcoal retained, to unite with the water to form from these substances in different proportions the solid wood, the gummy ferina, and the saccharine sap. Wonderful fecundity of nature? which can fashion from the same elements three different substances, and has power in addition to transmute them, one into another, with the least possible expenditure of force, as often as occasion demands the change.*

The following are among the closing thoughts of the address:—

"This ever-acting mutation of the atom is due in part to our air, which, containing so much oxygen loosely mingled in, readily yields that element to the first petitioner which presents itself, and it, like a Circassian beauty in the market, seems waiting for the offer, and anxious for the union. The brightest plate of metal is soon tarnished by it, and where it cannot corrode, it will film. It is the trouble of the daguerreotypist to keep his plate clean; he washes, he rubs, he dissolves, and he coats anew the surface occasionally, to keep a pure metallic surface; and when gotten, he has to shut it up from the light, for strange to tell, every ray of light lays its image there, unseen by our coarser gaze, but then needing only the more sensitive surface to make it plain to us.

"The slightest article which we leave out of our hands leaves its image where it rests. A copper coin upon a silver one—a silver coin upon a mirror, leave images which even our breath can make evident to our eye; and it is possible for us to take upon a warm copper ptate an image or copy of a newspaper.

"Nature does not merely condescend to paint the portrait or the landscape upon the coated plate in the atelier of the artist. She is no niggard in her pencil. Every where and at all times where there are rays of light wandering,—where there is a movement to be chronicled,—where there is, to speak technically, a ground to receive the painting, there the image is drawn, painted in an invisible ink. Wherever in crowds we gather within a building, wherever individuals may transact private business together, there is the angel of light tracing on the walls the images of the acts going on, whether they be good, or whether they be evil;—images invisible, ineffaceable; and, like as the manuscript of the middle ages, contain many works copied in one over the other,—those underneath not effaced, but only covered over, so as suitably to receive the more modern thoughts; so like leaves in a book, on these walls are there depicted the successive acts of individuals.

"With this power of the sun to record passing events, may we not understand, in a holier sense, the office of this luminary, who was set for us, "to rule the day;" and how awfully does the suggestion flit through our minds, that against every one of us there

* Dumas Org. Bal.

is a *handwriting of God recorded upon the wall*, which it needs little more than the learning of Egypt to expound and explore."

We thank Prof. Antisell for the pleasure we have enjoyed in the perusal of his tasteful and elegant address. Such lectures are worthy of all praise, and the young men to whom Dr. Antisell addressed this have shown that they are capable of appreciating true literary excellence in causing it to be published.

